# Supplementary material for: SARS-CoV-2 host-shutoff impacts innate NK cell functions, but antibody-dependent NK activity is strongly activated through non-spike antibodies
Source: eLife. 2022 May 19;11:e74489. doi: 10.7554/eLife.74489 (PMC9239683; doi:10.7554/eLife.74489)
Supplement: Supplementary file 2. [file elife-74489-supp2.docx]

**Table S2.** List of interferon inducible genes identified in the filtered PM dataset

B2M

BST2

CCRL2

CD47

CNP

CSF1

HLA-C

ISG15

LAP3

LGALS3BP

MOV10

MVB12A

MX1

OAS1

PARP14

PLSCR1

PROCR

PSMA3

PSME1

PSME2

RNF31

STAT2

WARS1
