# Supplementary material for: SARS-CoV-2 host-shutoff impacts innate NK cell functions, but antibody-dependent NK activity is strongly activated through non-spike antibodies
Source: eLife. 2022 May 19;11:e74489. doi: 10.7554/eLife.74489 (PMC9239683; doi:10.7554/eLife.74489)
Supplement: Supplementary file 3. [file elife-74489-supp3.docx]

**Table S3.** Clinical characteristics of patients giving longitudinal serum samples

| Study ID | Severity | Age | Gender | Past medical history |
| --- | --- | --- | --- | --- |
| 1 | **Mild** | **82** | **M** | **dementia, ESRF, HTN, IHD, PVD** |
| 2 | **Mild** | **77** | **F** | **ESRF, HTN, T2DM** |
| 3 | **Mild** | **64** | **F** | **AF, CVA, dementia, ESRF, HTN, T2DM** |
| 4 | **Mild** | **27** | **F** | **ESRF, obesity** |
| 5 | **Mild** | **48** | **F** | **ESRF, HTN, obesity** |
| 6 | **Severe** | **52** | **F** | **Asthma, obesity** |
| 7 | **Severe** | **62** | **M** | **colon ca, epilepsy, HTN, obesity, T2DM** |
| 8 | **Severe** | **65** | **F** | **HTN, hypothyroidism, obesity** |
| 9 | **Severe** | **61** | **M** | **ESRF, obesity, VTE** |
| 10 | **Severe** | **57** | **M** |  |
| 11 | **Mild** | **23** | **F** | **CKD, HTN, T1DM** |
| 12 | **Mild** | **62** | **M** | **ESRF, HTN, renal transplant** |
| 13 | **Mild** | **25** | **F** | **AML** |
| 14 | **Mild** | **52** | **M** | **CVA, prostate ca, T2DM** |
| 15 | **Mild** | **88** | **F** | **AF, CCF, CKD, COPD, IHD, RA, T2DM** |
| 16 | **Mild** | **64** | **M** |  |
| 17 | **Mild** | **69** | **M** | **AF, HTN, T2DM, VTE** |
| 18 | **Mild** | **22** | **M** | **NHL** |
| 19 | **Severe** | **49** | **M** |  |
| 20 | **Severe** | **67** | **F** | **AF, HTN, obesity, T2DM** |

AF=atrial fibrillation, AML=acute myeloid leukaemia, ca=cancer, CCF=congestive cardiac failure, CKD=chronic kidney disease, COPD=chronic obstructive pulmonary disease, CVA cerebrovascular accident, ESRF=end-stage renal failure, HTN=hypertension, IHD=ischaemic heart disease, NHL=non-Hodgkin lymphoma, RA=rheumatoid arthritis, T1DM/T2DM=type 1/2 diabetes mellitus, VTE=venous thromboembolism.
