# Supplementary material for: SARS-CoV-2 host-shutoff impacts innate NK cell functions, but antibody-dependent NK activity is strongly activated through non-spike antibodies
Source: eLife. 2022 May 19;11:e74489. doi: 10.7554/eLife.74489 (PMC9239683; doi:10.7554/eLife.74489)
Supplement: Supplementary file 4. [file elife-74489-supp4.docx]

| **mAb name** | **Specificity** | **Competition group** | **S EC50** | **RBD EC50** | **NTD EC50** | **S2 ELISA** | **SARS-CoV-2 WT FL IC50** | **SARS-CoV-2 WT PV IC50** |
| --- | --- | --- | --- | --- | --- | --- | --- | --- |
| P008_060 | SD1 | 7 | 0.031 | >10 | >10 | n.d. | 32.90 | 0.095 |
| P008_004 | S2 | n.d. | 0.045 | >10 | >10 | + | >100 | >100 |
| P008_005 | S2 | n.d. | 0.20 | >10 | >10 | + | >100 | >100 |
| P008_006 | S2 | n.d. | 0.10 | >10 | >10 | + | >100 | >100 |
| P008_016 | S2 | n.d. | 0.11 | >10 | >10 | + | >100 | >100 |
| P008_023 | S2 | n.d. | 0.40 | >10 | >10 | + | >100 | >100 |
| P008_032 | S2 | n.d. | 0.060 | >10 | >10 | + | >100 | >100 |
| P008_051 | NTD | 5 | 0.091 | >10 | 0.056 | n.d. | 48.82 | 0.30 |
| P008_052 | NTD | 5 | 0.085 | >10 | 0.063 | n.d. | 48.65 | 0.13 |
| P003_027 | NTD | 6 | 0.0039 | >10 | 0.0065 | n.d. | 1.64 | 12.92 |
| P008_056 | NTD | 6 | 0.060 | >10 | 0.049 | n.d. | 0.014 | >100 |
| P008_001 | NTD | n.d. | 0.82 | >20 | 1.20 | n.d. | >100 | >100 |
| P008_002 | NTD | n.d. | 0.0084 | >20 | 0.015 | n.d. | >100 | >100 |
| P008_014 | NTD | n.d. | 0.066 | >20 | 0.021 | n.d. | 1.81 | >100 |
| P008_099 | NTD | n.d. | 0.022 | >20 | 0.15 | n.d. | >100 | >100 |
| P054_044 | NTD | n.d. | 0.031 | >20 | 4.00 | n.d. | 0.94 | 0.74 |
| P003_016 | NTD | n.d. | 0.13 | >20 | 2.00 | n.d. | 0.15 | 0.45 |
| P003_014 | RBD | 1 | 0.0076 | 0.0086 | n.d. | n.d. | >100 | >100 |
| P054_027 | RBD | 1 | 0.0077 | 0.013 | n.d. | n.d. | >100 | 0.7 |
| P008_018 | RBD | 3 | 0.071 | 0.082 | n.d. | n.d. | 0.055 | 0.00081 |
| P008_090 | RBD | 3 | 0.018 | 0.026 | n.d. | n.d. | 0.071 | 0.0042 |
| P008_108 | RBD | 3 | 0.0092 | 0.014 | n.d. | n.d. | 0.0023 | 0.0031 |
| P008_015 | RBD | 4 | 0.034 | 0.036 | n.d. | n.d. | 0.055 | 0.00012 |
| P008_096 | RBD | 4 | 0.030 | 0.055 | n.d. | n.d. | 9.09 | 0.41 |

Table S4. Monoclonal Ant-spike Antibodies used, data taken from^51^
