# Supplementary material for: SARS-CoV-2 host-shutoff impacts innate NK cell functions, but antibody-dependent NK activity is strongly activated through non-spike antibodies
Source: eLife. 2022 May 19;11:e74489. doi: 10.7554/eLife.74489 (PMC9239683; doi:10.7554/eLife.74489)

# Uncropped Blot From Fig. 3B

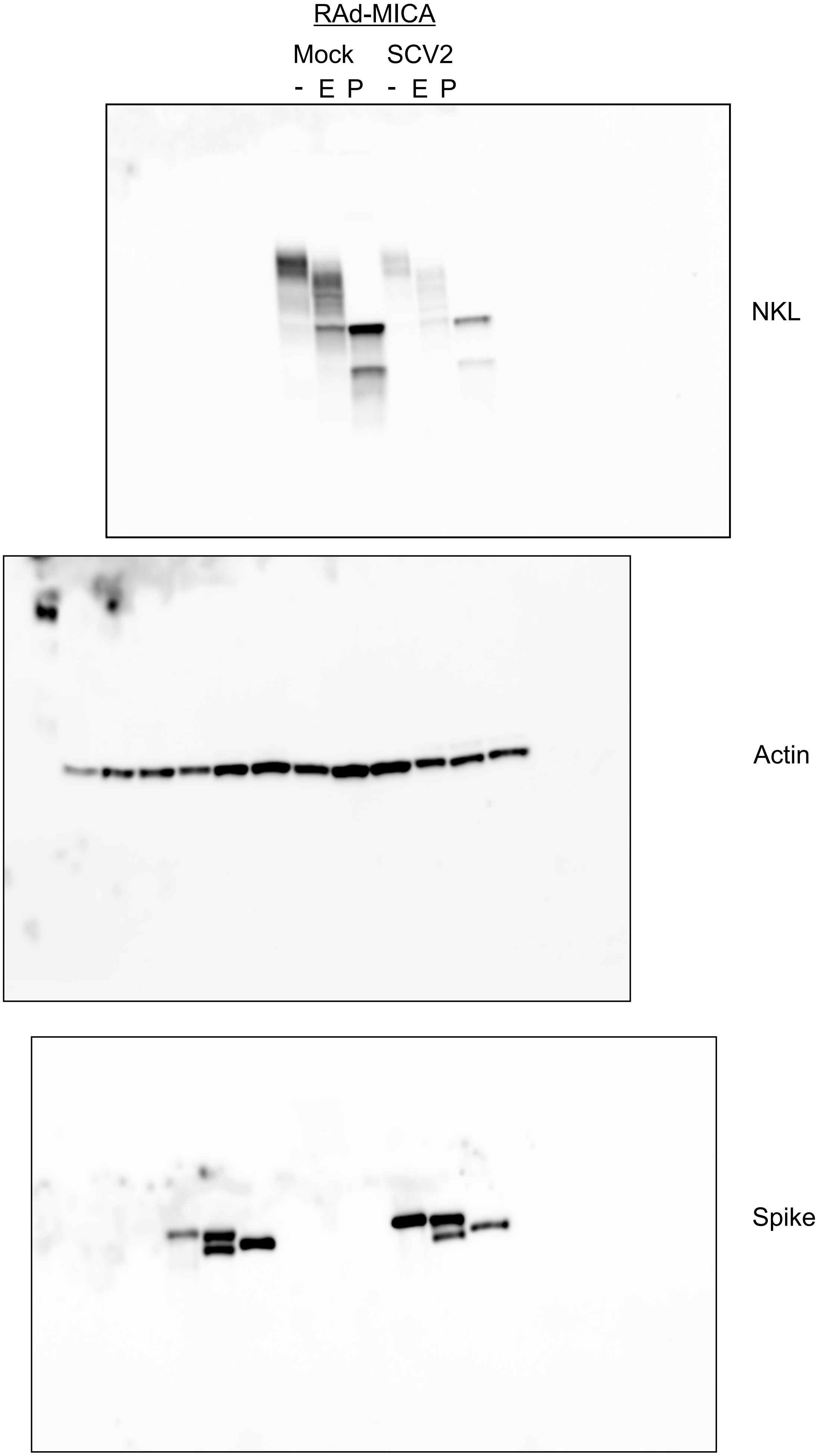

# Uncropped Blot From Fig. 3B

RAd-ULBP2

Mock SCV2

- E P - E P

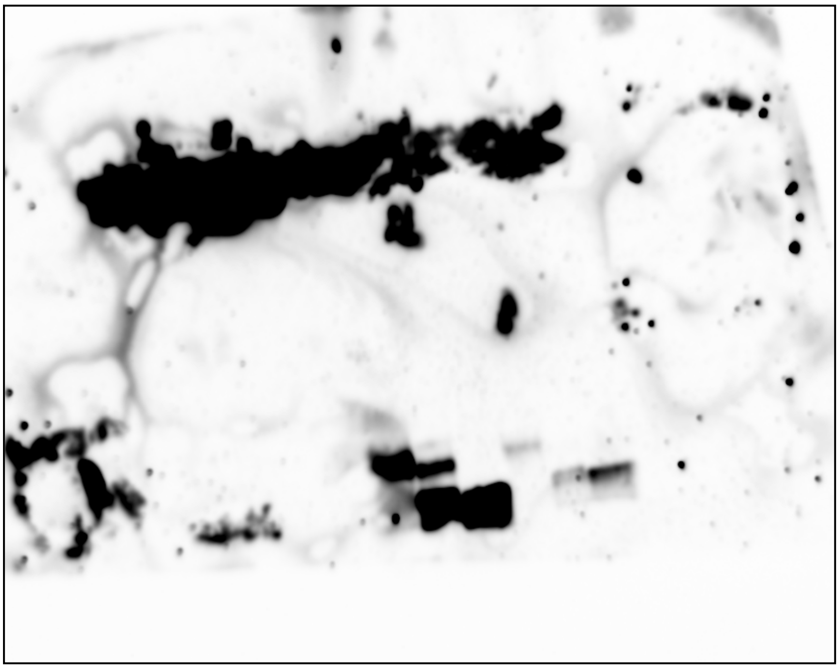

NKL

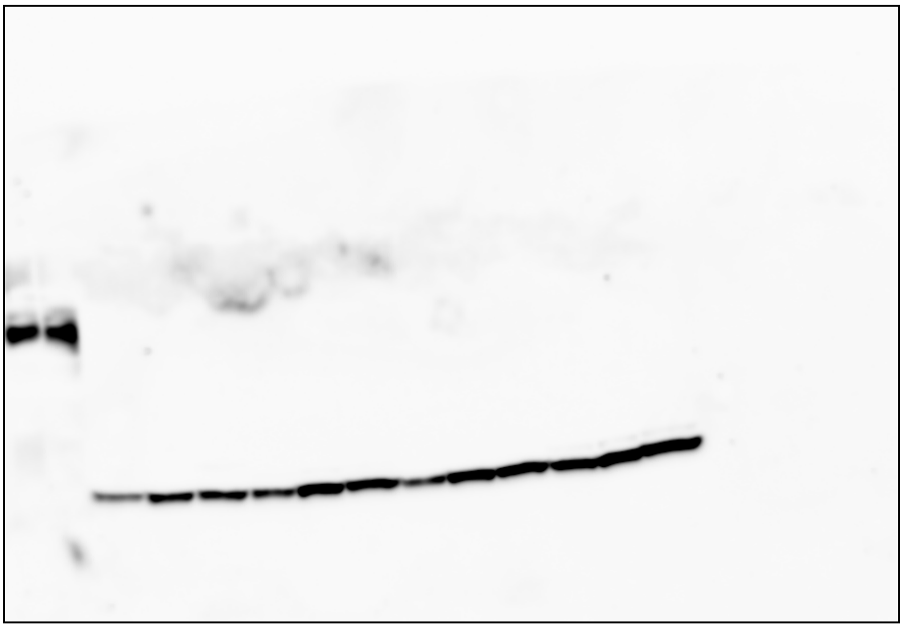

Actin

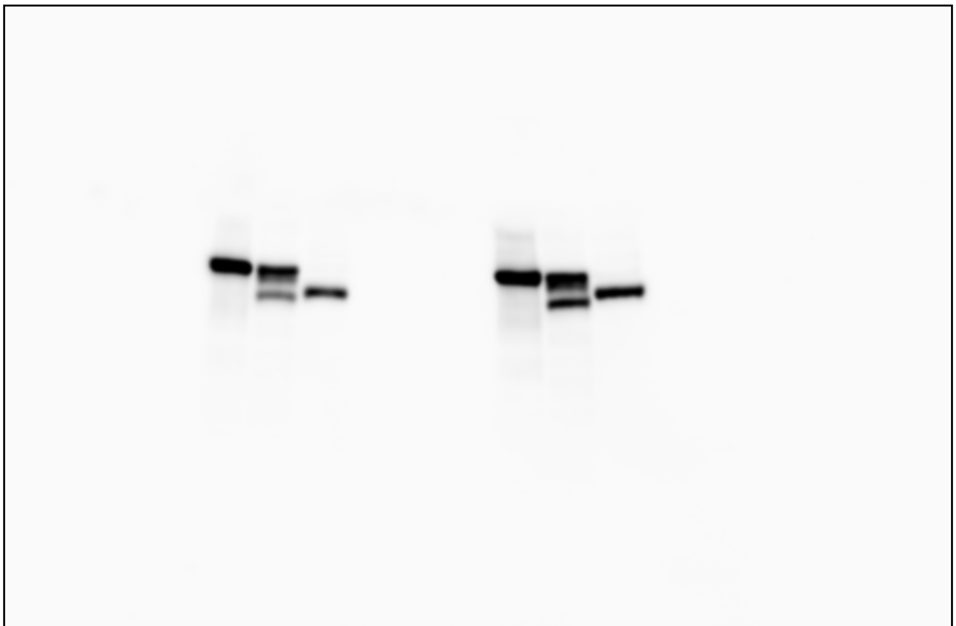

Spike

# Uncropped Blot From Fig. 3B

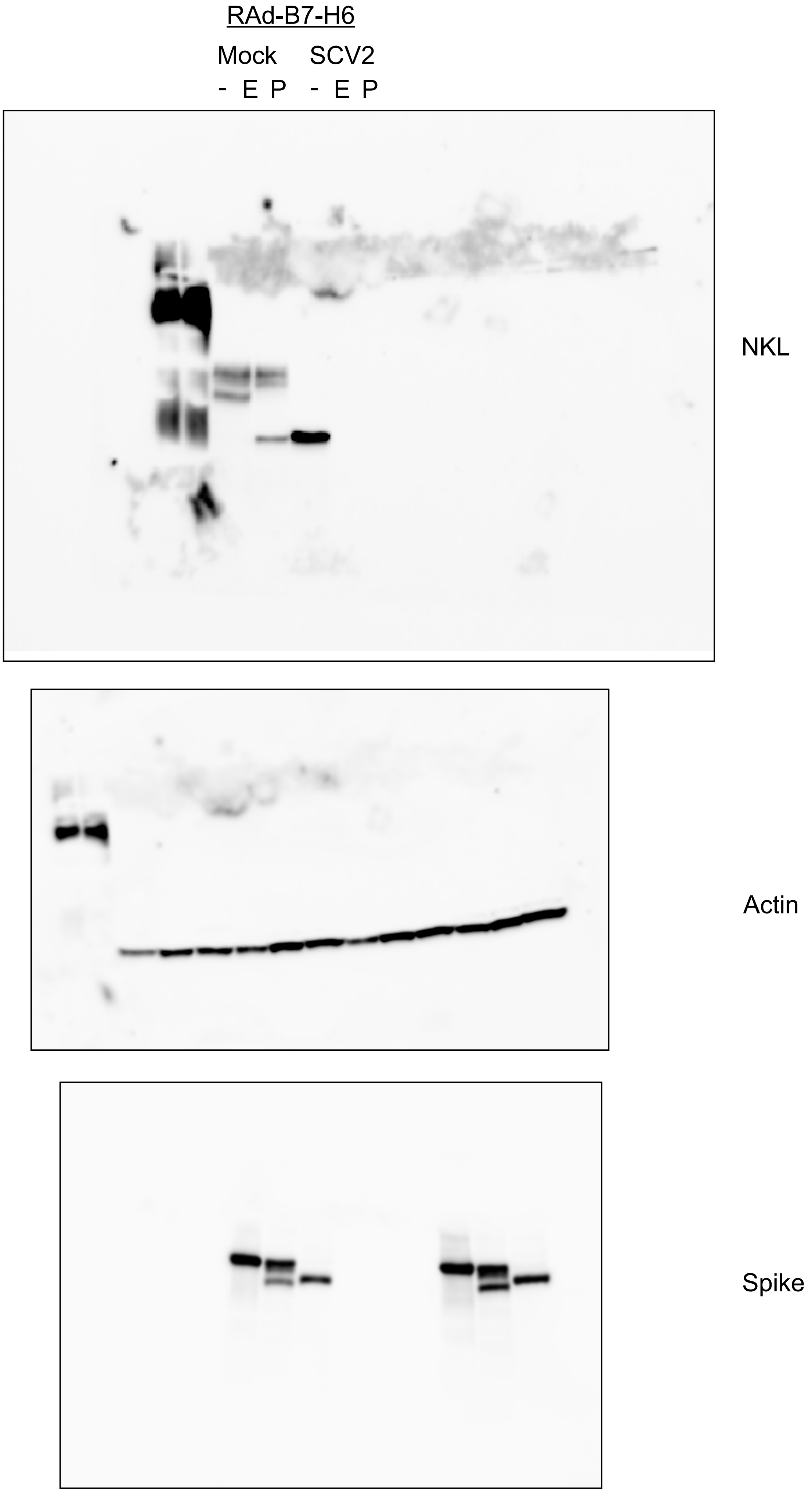

# Uncropped Blot From Fig. 3C

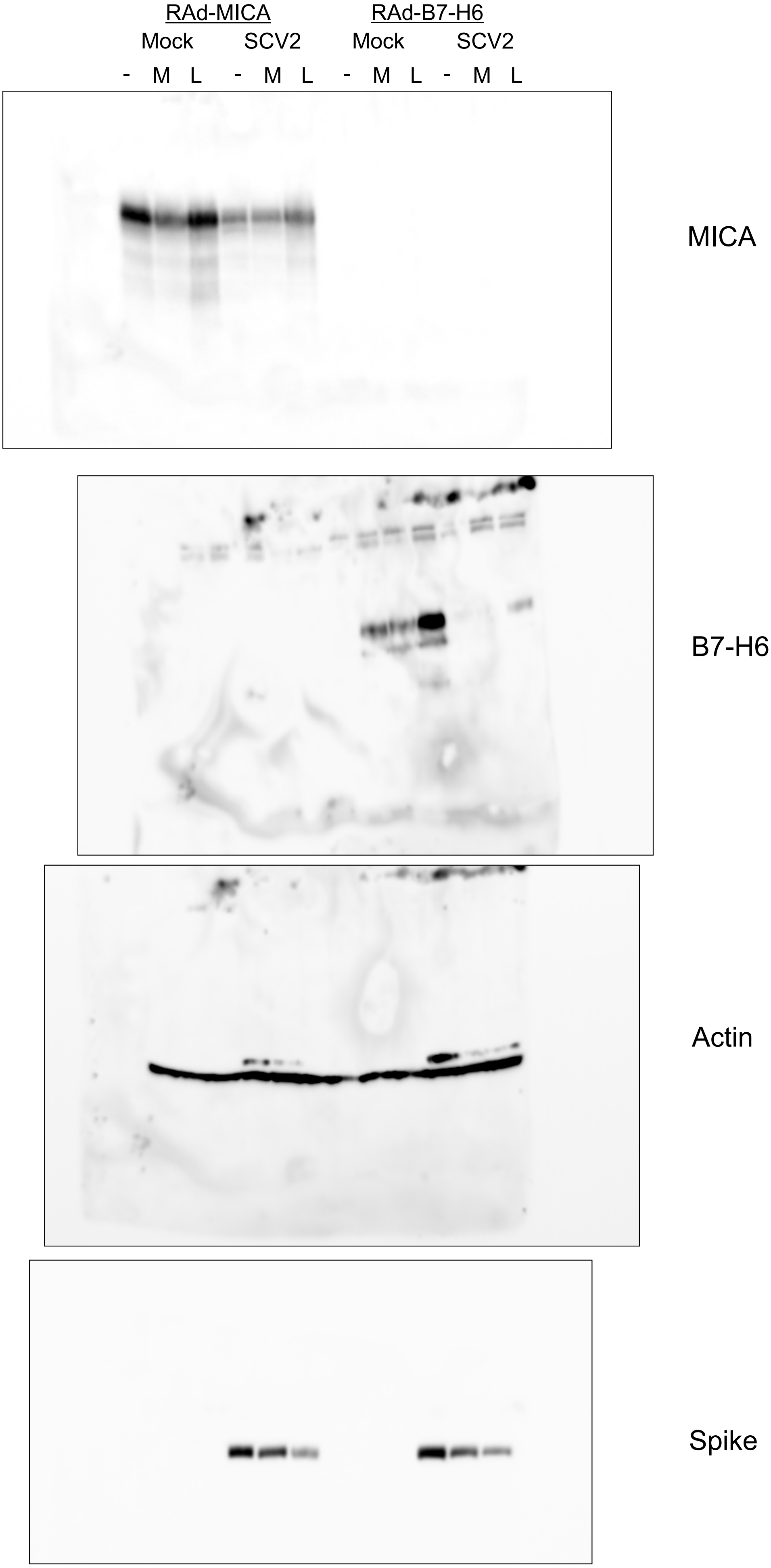

# Uncropped Blot From Fig. 4A

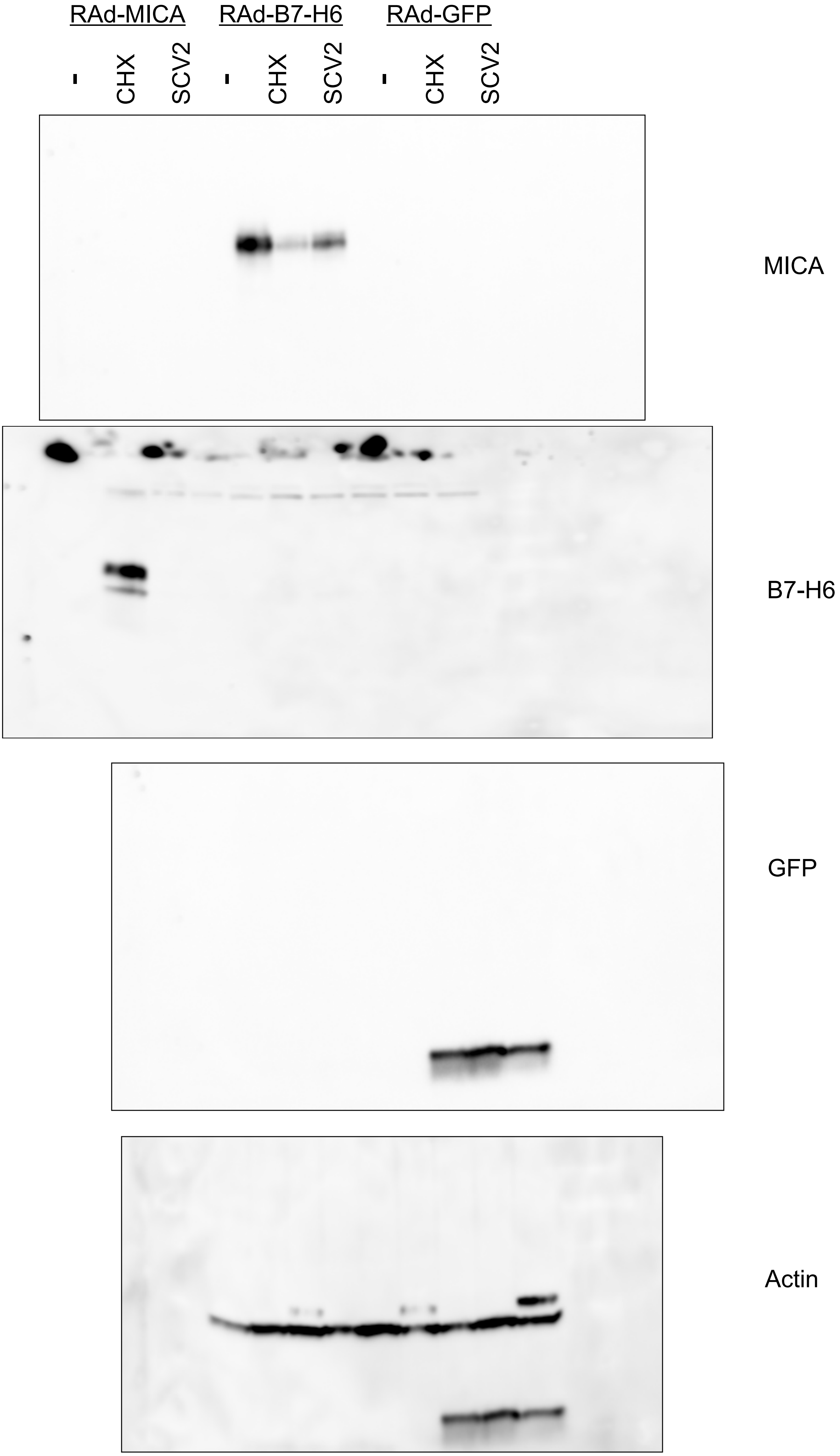

Supplement: Source data 1. — Raw files for Figure 3B are provided as follows: Data 1 = MICA, Data 2 = Actin, Data 3 = Spike (all samples from RAd-MICA experiment). Data 4 = ULBP2, Data 5 = Actin, Data 6 = Spike (all samples from RAd-ULBP2 experiment). Data 7 = B7-H6, Data 8 = Actin, Data 9 = Spike (all samples from RAd-B7-H6 experiment). Raw files for Figure 3C are provided as follows: Data 1 = MICA, Data 2 = B7-H6, Data 3 = Actin, Data 4 = Spike Raw Files for Figure 4A are provided as follows: Data 1 = MICA, Data 2 = B7-H6, Data 3 = GFP, Data 4 = Actin [file elife-74489-data1.zip › Uncropped Blots.pdf]
